# New mutation of CACNA1H p.Tyr613Phe in hyperaldosteronism: a case report

**DOI:** 10.3389/fmed.2025.1715935

**Published:** 2026-01-12

**Authors:** Qing Yan, Xinyi Qu, Rong Wang, Wenxuan Ji, Li Li, Qingqing Bi

**Affiliations:** 1 Department of Clinical Laboratory, Qingdao Central Hospital, University of Health and Rehabilitation Sciences, Qingdao, China; 2 Department of Nephrology, Qingdao Central Hospital, University of Health and Rehabilitation Sciences, Qingdao, China

**Keywords:** aldosteronism, CACNA1H, familial hyperaldosteronism, mutation, p.Tyr613Phe

## Abstract

**Background:**

Primary aldosteronism (PA) is an endocrine disorder characterized by the autonomous, excessive production of aldosterone from the adrenal glands. Familial hyperaldosteronism (FH) is one type of PA. FH is further subclassified into types I through IV according to different gene mutations.

**Case presentation:**

This paper reports a case of unilateral adrenal hyperplasia with germline CACNA1H mutation, p.His515Tyr and p.Tyr613Phe, confirmed by endocrine test, whole exome sequencing and Sanger sequencing 4 years after onset. The variants located within N-terminal close to the first transmembrane domain of the protein that was highly conserved across different species. Polyphen2 and PROVEAN predicted p.Tyr613Phe to be probably damaging and deleterious.

**Conclusion:**

These findings broaden the genetic spectrum of PA and offer novel insights into the molecular mechanisms driving excessive aldosterone production. Therefore, genetic sequencing is recommended for PA patients whose etiology remains unclear after standard clinical evaluation.

## Introduction

Primary aldosteronism is caused by the autonomous and excessive secretion of aldosterone, mainly characterized by secondary hypertension and hypokalemia, with a prevalence of 5 to 10% (1). Usually, PA is classified to: bilateral adrenal hyperplasia (about 60% of cases), aldosterone-producing adenomas (APAs; about 30% of cases), unilateral adrenal hyperplasia, malignancy, and familial hyperaldosteronism (FH) (2). The underlying causes of PA involve germline and somatic mutations that lead to dysregulated aldosterone production. Germline mutations play a significant role in the development of familial hyperaldosteronism. Mutations in KCNJ5, CACNA1D, ATP1A1 and ATP2B3 genes, have been identified in at least 50% of patients with excess aldosterone production (3, 4). These mutations lead to excessive aldosterone production and are responsible for a subset of PA cases. All these mentioned genetic mutations increase intracellular calcium levels as a signal and, as a result, trigger the expression of CYP11B2 to form aldosterone synthase, which catalyzes aldosterone biosynthesis (5).

Calcium voltage-gated channel subunit alpha1 H, short for CACNA1H, encodes the T-type voltage-gated calcium channel Ca_V_3.2, which is an important component of electrical and intracellular calcium oscillations and govern aldosterone production. Germline gain-of-function mutations in CACNA1H were identified in familial hyperaldosteronism type IV. CACNA1H p.M1549V was found in children with early-onset PA via autosomal dominant transmission, as a cause of FH-IV (6). Other germline CACNA1H variants, such as p.M1549I, p.S196L, and p.P2083L, were also found in patients with FH-IV (7). Besides, a few studies reported somatic CACNA1H mutations, such as p.I1430T (8) and p.V1937M (9), causing APAs.

The damage degree of hypertension target organs such as heart and kidney in PA patients is more serious than that in essential hypertension patients, and early diagnosis and treatment is particularly important. This paper reports a case of unilateral adrenal hyperplasia with germline CACNA1H mutation, p.His515Tyr and p.Tyr613Phe, confirmed by endocrine test and gene sequencing 4 years after onset. The characteristics of this case were summarized and analyzed in order to provide clinical data for the subsequent genetic research of this disease, and to provide ideas for clinicians to make clear diagnosis of this disease.

## Case presentation

In June 2019, a 29-year-old male was admitted to our outpatient department complaining of a 3-month history of headache, dizziness, and blurred vision. The patient had received no specific treatment prior to that visit. His consulting room measured blood pressure was 220/123 mmHg on the left upper arm and 240/152 mmHg on the right upper arm. After hospitalization, routine laboratory examinations were performed.

**Figure 1:**
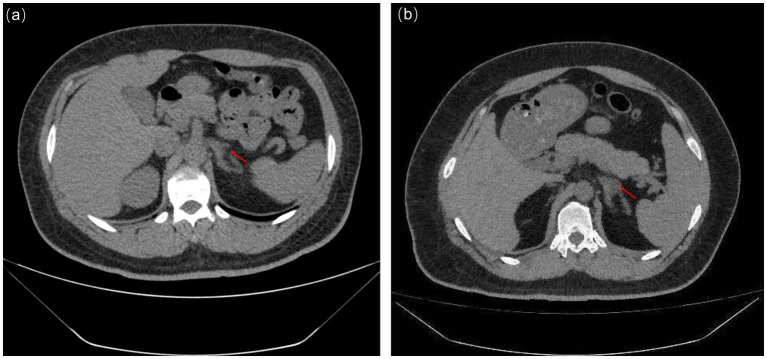
CT scan of left adrenal gland in June, 2019 **(a)** and September, 2023 **(b)**.

Potassium: 2.8 mmol/L (3.5–5.3), urea: 17.6 mmol/L, creatinine: 571 μmol/L, parathyroid hormone (PTH): 251.6 pg/mL, aldosterone (June 2019): 429.78 pg/mL, renin (June 2019): 22.97 pg/mL, aldosterone/renin (ARR): 18.71. More abnormal biochemical findings are detailed in Table 1. The computed tomography (CT) scan in 2019 revealed only very mild left adrenal thickening without obvious hyperplasia. In contrast, the follow-up CT in 2023 demonstrated a more pronounced structural change consistent with hyperplasia, as presented in Figure 1. The adrenal CT in 2023 revealed a nodule in the left adrenal gland with a diameter of 14 mm and an attenuation of 24 Hounsfield units (HU). In summary, the patient had hypokalemia with hypertension and elevated aldosterone, but the ARR ratio and imaging examination did not support the diagnosis of primary hyperaldosteronism and adrenal adenoma. Symptomatic treatment was immediately carried out. The treatment was primarily symptomatic, focusing on controlling blood pressure, reducing creatinine levels, and protecting kidney function.

**Table 1:** The main laboratory results in the patient with hyperaldosteronism.

Laboratory examination	Patient value		Reference range
	June 2019	September 2023	
Potassium (mmol/L)	2.8	4.0	3.5–5.3
Urea (mmol/L)	17.6	39.7	3.1–8.0
Creatinine (μmol/L)	571	1,121	57–97
24 h urine protein (g)	2.82	4.95	0–0.15
PTH (pg/mL)	251.60	191	15–75
B-type natriuretic peptide (ng/mL)	162.63	35.82	0–100
Troponin I (ng/mL)	0.13	0.02	0–0.06
Aldosterone (pg/mL)	429.78	998.48	31–351
Renin (pg/mL)	22.97	9.16	4.7–47.6
Aldosterone/Renin (ARR)	18.71	109	0–37
ACTH (pg/mL)	44.66	63.48	7.0–65.0
Cortisol (μg/dL)	17.90	17.63	6.4–22.8

Table 1 also presents patient’s data of laboratory tests in September 2023, showing a continuous increase in aldosterone, positive ARR, improved cardiac function and continuous deterioration of renal function. A seated, morning aldosterone-to-renin ratio (ARR) of >30 is used as the screening cutoff for PA, following the Endocrine Society Clinical Practice Guideline. The patient was unable to tolerate adrenal venous sampling (AVS) due to consistently poor renal function. Then whole exome sequencing was initiated due to a confluence of factors that deviated from a typical sporadic case: the patient’s young age at presentation, the definitive biochemical diagnosis (hypertension, hypokalemia, elevated ARR) and the inability to perform AVS. Therefore, WES was not performed routinely but was specifically indicated to investigate a potential hereditary cause, which could critically impact clinical management.

Library construction, WES and data analyses were carried out by Shanxi Guoxin Caregeno Biotechnology Co., Ltd., China. Genomic DNA was extracted using a TIANamp Blood DNA Kit (Tiangen Biotech Co. Ltd., Beijing, China) from whole blood and stored at −80 °C. According to the manufacturer’s protocol, 200 ng genomic DNA of each individual was sheared by Biorupter (Diagenode, Belgium) to acquire 150–200 bp fragments. The ends of DNA fragment were repaired and Illumina Adaptor was added (Fast Library Prep Kit, iGeneTech, Beijing, China). After sequencing library were constructed, the whole exons were captured using AIExome^®^ Human Exome Panel V3 with TargetSeq One^®^ Hyb & Wash Kit v2.0 (iGeneTech, Beijing, China) and sequenced on Illumina platform (Illumina, San Diego, CA) with 150 base paired-end reads. Raw reads were filtered to remove low quality reads by using FastQC. Then clean reads were mapped to the reference genome GRCh38 by using Bwa. After removing duplications, SNV and InDel were called and annotated by using GATK.

Sanger sequencing was performed further. We directly sequenced the PCR products to genotype the CACNA1H c.1838A>T and CACNA1H c.1543C>T polymorphisms in the remaining individuals. The primers (CACNA1H c.1838A>T-F: 5′-GGCCACATATTCCGCAAGGTCA-3′, CACNA1H c.1838A>T-R: 5′-CGCGCCAGCTCGGACCAG-3′; CACNA1H-EX9-F: ACCATGCCGACTGCCACATAGAGG, CACNA1H-EX9-R: CTCACCATGCTCCCCGACCAC) were designed using the Primer 5.0 software (PREMIER Biosoft International, Palo Alto, CA, United States). The reactions were performed in 20 μL volume containing 50 ng template DNA, 10 μL 2×HieffTm PCR Master Mix (YESEN, Shanghai, China), 10 pmol forward primers, 10 pmol reverse primers. Then PCR amplification was performed under the following conditions: a pre-denaturation cycle at 94 °C for 4 min; 35 cycles at 94 °C for 20 s, 55 °C for 20 s, 72 °C for 1 min; and a final extension cycle at 72 °C for 10 min. The obtained products were cooled to 4 °C. The PCR products were directly sequenced.

For the predictions of sequence variants, computational (*in silico*) programs of Combined Annotation Dependent Depletion (CADD, v1.3) (10), Polymorphism Phenotyping v2 (PolyPhen-2 v2.2.2 build r394) (11) and Protein Variation Effect Analyzer (PROVEAN) (12) were applied. The combined prediction results, without failing more than one of the three *in silico* tools, would be considered as a single piece of evidence supporting a deleterious effect from a variant. Visualization of CACNA1H protein with the mutated sequence was performed by a Protter tool (13).

**Figure 2:**
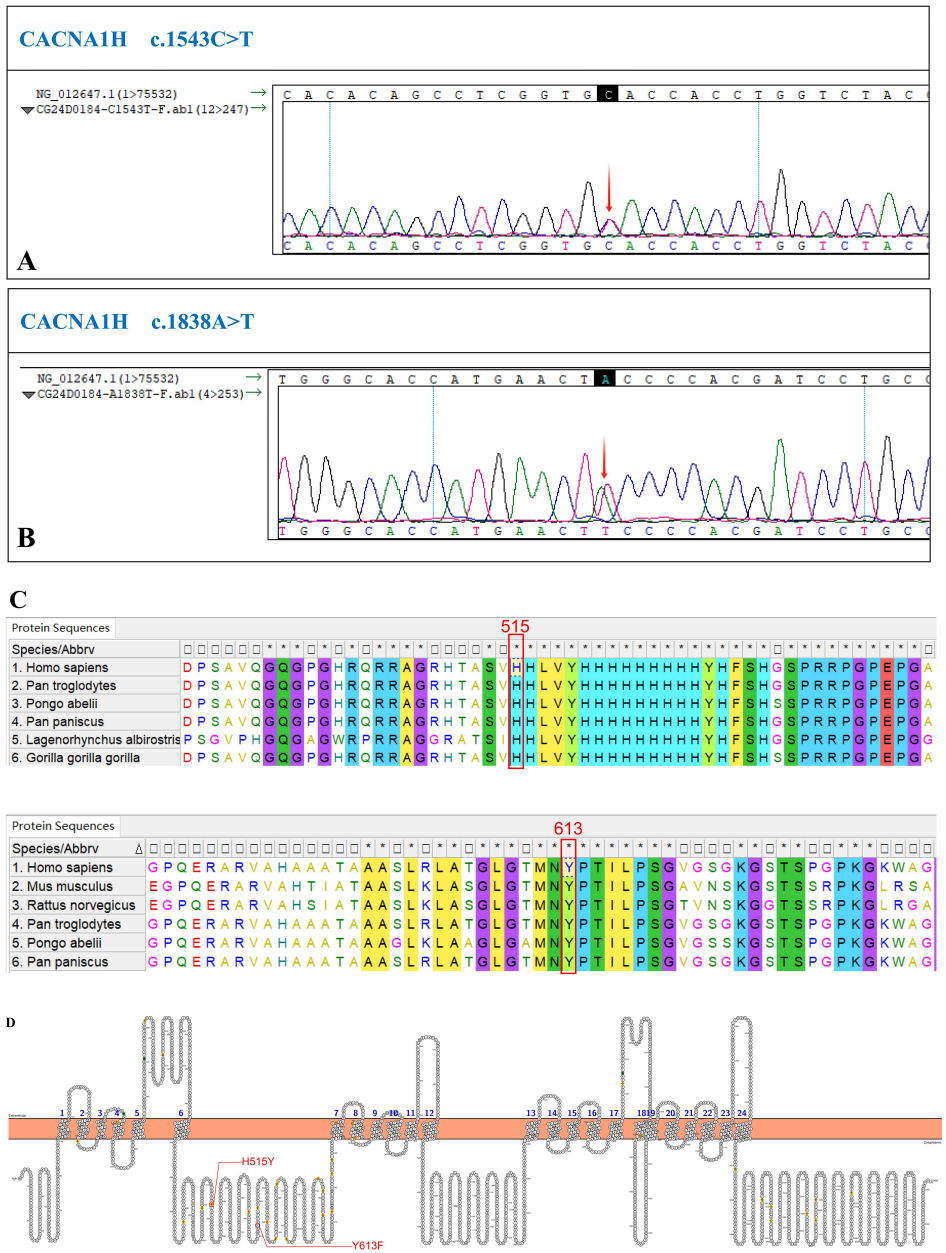
Identification of a new *CACNA1H* variant. **(A)** The sequencing chromatogram of variant c.1543C>T. **(B)** The sequencing chromatogram of variant c.1838A>T. **(C)** Conservation of these mutant amino acids among different species. **(D)** The structure of human CACNA1H Ca_V_3.2 calcium channel. The red lines indicate the replaced amino acids identified in this study, which is located in the N-terminal domain.

We performed a targeted gene panel next-generation sequencing on DNA extracted from peripheral blood for mutations which revealed two uncommon variants in CACNA1H, c.1543C>T (p.His515Tyr) and c.1838A>T (p.Tyr613Phe) (Table 2; Figures 2A,B). The sequence was changed by single nucleotide mutations in *CACNA1H*, which encodes calcium voltage-gated channel subunit alpha1 H.

**Table 2:** Results of whole exome sequencing.

Gene	Chr	Nucleic acid variants	Amino acid change	Mutation types	Genotypes	ACMG classification
CACNA1H	16	c.1543C>T	p.His515Tyr	Missense mutation	Heterozygous mutation	Uncertain significance
CACNA1H	16	c.1838A>T	p.Tyr613Phe	Missense mutation	Heterozygous mutation	Likely pathogenic

Chr, chromosome; ACMG, American College of Medical Genetics and Genomics.

According to the evolutionary conservation analysis, amino acids H515 and Y613 in wild-type CACNA1H are conserved across different species (Figure 2C). The replaced amino acid of CACNA1H p.Tyr613Phe lies in the N-terminal domain close to the first transmembrane segment of the channel (Figure 2D).

The variants were present in the patient, but his parents refused genetic testing. The variants were located in the N-terminal region, close to the first transmembrane domain of the protein. This region is highly conserved across species, suggesting that the variants are likely deleterious.

The p.His515Tyr variant has been recorded in ClinVar.^1^ The p.Tyr613Phe variant has not been recorded in several databases, including ClinVar, PubMed and Human Gene Mutation Database (HGMD, https://www.hgmd.cf.ac.uk/), indicating that this variant is novel.

To evaluate the pathogenicity of the newly identified variant, p.Tyr613Phe, several prediction tools were used. In Polyphen2, this mutation was predicted to be probably damaging with a score of 0.991. Besides, PROVEAN predicted the variant to be deleterious with a score of −3.163.

**Figure 3:**
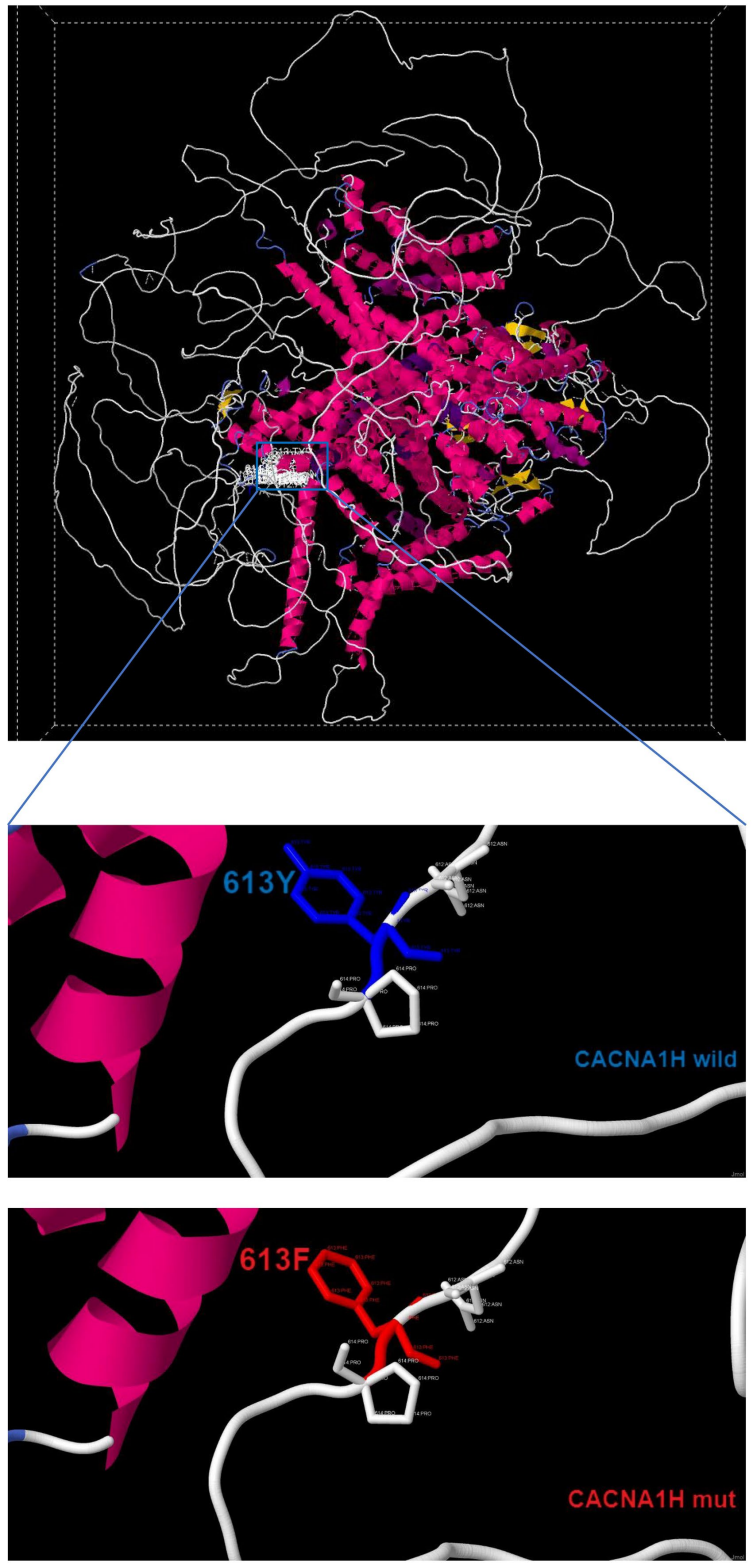
Three-dimensional structure of wild-type CACNA1H and mutant-type CACNA1H.

The prediction of protein 3D structure revealed that Tyr613 residue locates near an a-helix, and p.Tyr613Phe causes a change from hydrophobic amino acids to amphoteric amino acids, which may lead to alterations in protein conformation and stability (Figure 3). According to the ACMG guidelines, the classification for p.Tyr613Phe was likely pathogenic (PM2 + PP3 + PP4) (14).

The patient was managed with a complex antihypertensive regimen initiated in June 2019 that specifically included the mineralocorticoid receptor antagonist spironolactone, alongside calcium channel blockers, beta-blockers, and diuretics. This treatment led to a significant improvement in blood pressure, from a malignant range (up to 240/152 mmHg) to a controlled range (130–150/80–100 mmHg). Key clinical trends were observed: persistent hypokalemia showed partial correction with therapy, and renal function remarkably improved, with serum creatinine decreasing from an acute peak of 609 μmol/L to a stable chronic level of 190–245 μmol/L. The patient’s positive clinical and biochemical response to the MRA-based regimen provides compelling indirect evidence of a pathophysiological process involving aldosterone pathway dysregulation, thereby enhancing the clinical relevance of the CACNA1H variants discovered.

## Discussion

PA is a prevalent cause of secondary hypertension. It often remains undiagnosed in a timely and accurate fashion owing to a multitude of factors. A Chinese epidemiological investigation has revealed that the prevalence of PA among patients with hypertension exceeds 4% (15). Evidently, PA represents a prevalent but frequently underdiagnosed condition. When compared with patients suffering from essential hypertension, those with PA exhibit more severe hypertension-related target organ damage. Therefore, early diagnosis and treatment are of particular significance. ARR is a crucial biomarker in the diagnosis of primary aldosteronism. However, in this case, the initial ARR fell below this threshold, complicating the definitive diagnosis of PA at an early stage. As the disease progressed, however, a substantial increase in ARR was observed. This dynamic fluctuation in ARR may be attributed to several factors. Notably, the use of medications such as spironolactone and metoprolol during treatment may reversibly elevate the ARR. Additionally, the presence of impaired renal function itself may contribute to an increase in ARR. Confirmatory tests such as saline infusion or captopril challenge were not performed due to the patient’s comorbid renal insufficiency and hypertensive heart disease which presented relative contraindications for these procedures. CT plays an important role in the diagnosis of PA and in excluding microadenoma. Microadenomas are often characterized by more pronounced contrast enhancement in the arterial phase and typically exhibit rapid contrast washout in the venous phase. In contrast, the hyperplastic nodule in our case was described to enhance similarly to the surrounding normal adrenal tissue, showing minimal significant enhancement. It is stated that, in the absence of histopathological confirmation, this distinction remains a radiological interpretation based on these imaging characteristics. The typical clinical manifestations of PA encompass hypertension and hypokalemia, with hypertension being the most frequently encountered symptom. In certain patients, serum potassium levels may be normal. PA can be classified into various types according to its etiology. Familial hyperaldosteronism (FH) is one such type. Currently, FH is further subclassified into types I through IV, all of which are monogenic autosomal dominant genetic disease.

In this study, we identified a novel germline mutation of CACNA1H p.Tyr613Phe associated with PA through whole exome sequencing and confirmed by targeted sequencing. The identified missense mutation is located near an a-helix and the variant which may lead to alterations in protein conformation and stability, consistent with the hyperaldosteronism phenotype observed in patients. Notably, the mutation’s absence in population databases (e.g., gnomAD, ClinVar) indicates CACNA1H p.Tyr613Phe is new variant associated with FH. Currently, FH is linked to mutations in genes such as CYP11B1/CYP11B2 (FH-I), CLCN2 (FH-II), KCNJ5 (FH-III), which regulate adrenal cell membrane potential or calcium signaling. In this case, no pathogenic mutations were found in other FH-related genes. Our discovery of CACNA1H p.Tyr613Phe locates within N-terminal close to the first transmembrane domain of the protein that was highly conserved across different species. Furthermore, p.Tyr613Phe causes a change from hydrophobic amino acids to amphoteric amino acids, which may lead to alterations in protein conformation and stability. The p.His515Tyr variant has been recorded in ClinVar (SCV001035489) and considered to be benign. Unlike somatic mutations often found in aldosterone-producing adenomas, this germline variant suggests a heritable form of PA, emphasizing the need for genetic screening in familial or early-onset cases. The identification of this mutation may enable personalized management. For instance, predictive genetic testing may be recommended for first-degree relatives. For identified carriers, regular biochemical screening can be initiated to detect hyperaldosteronism early, and unilateral adrenalectomy can be considered as a curative option before the onset of hypertension or its associated organ damages.

While our study establishes a strong association, larger cohorts are needed to validate the mutation’s prevalence across ethnicities. An additional limitation concerns the genetic analysis of the identified variant. Due to the refusal of the proband’s parents to undergo genetic testing, we were unable to perform familial segregation analysis. This precludes a conclusive determination of whether the variant is *de novo* or inherited. It is also important to note that the findings and conclusions of this study are primarily derived from integrated bioinformatics analysis and retrospective clinical observations. While these approaches have provided valuable insights into the potential association between p.Tyr613Phe and PA, a key limitation lies in the absence of direct functional validation. Specifically, the causal relationships and underlying molecular mechanisms suggested by our computational models have not been experimentally confirmed through *in vitro* or *in vivo* functional studies. Further experimental validation is essential to confirm the diagnostic and pathogenic roles of these candidates in PA. Mechanistic studies (e.g., adrenal-specific knockout models) could elucidate how this mutation disrupts aldosterone regulation. Furthermore, investigating interactions between this variant and environmental factor, such as dietary sodium intake, could help clarify its variable penetrance. The identification of the p.Tyr613Phe variant enhances our understanding of PA pathogenesis and creates new opportunities for precision medicine. Incorporating genetic testing into routine clinical practice may improve both diagnosis and targeted therapy for PA patients carrying this mutation. These findings broaden the known genetic spectrum of PA and offer novel insights into the molecular mechanisms driving excessive aldosterone production.

## Data Availability

The datasets presented in this study can be found in online repositories. The names of the repository/repositories and accession number(s) can be found at: https://www.ncbi.nlm.nih.gov/, SCV006336813.

## Footnotes

1
https://www.ncbi.nlm.nih.gov/clinvar/
